# Adipose-derived stem cells applied to ankle pathologies: a systematic review

**DOI:** 10.1007/s12306-023-00798-7

**Published:** 2023-11-09

**Authors:** A. Arceri, A. Mazzotti, E. Artioli, S. O. Zielli, F. Barile, M. Manzetti, G. Viroli, A. Ruffilli, C. Faldini

**Affiliations:** 1 1st Orthopaedics and Traumatologic Clinic, IRCCS Istituto Ortopedico Rizzoli, Via Giulio Cesare Pupilli 1, 40136 Bologna, Italy; 2 Department of Biomedical and Neuromotor Sciences (DIBINEM), Alma Mater Studiorum University of Bologna, 40123 Bologna, Italy

**Keywords:** Adipose derived stem cells, Ankle, Talus, Osteoarthritis, Osteochondral lesions

## Abstract

**Abstract:**

The purpose of this systematic review was to analyze the current use of adipose-derived mesenchymal stem cells (ADMSCs) and present the available evidence on their therapeutic potential in the treatment of ankle orthopedic issues, evaluating the applications and results. A literature search of PubMed, Google Scholar, EMBASE and Cochrane Library database was performed. The review was conducted following PRISMA guidelines. Risk of bias assessment was conducted through the Methodological Index for Non-Randomized Studies (MINORS) criteria. Initial search results yielded 4348 articles. A total of 8 articles were included in the review process. No clinical evidence has demonstrated the effectiveness of one isolation method over the other, but nonenzymatic mechanical method has more advantages. In all studies included significant clinical outcomes improvement were recorded in patients affected by osteochondral lesion and osteoarthritis of ankle. All studies performed a concomitant procedure. No serious complications were reported. ADMSC injection, especially through the nonenzymatic mechanical methods, looks to be simple and promising treatment for osteochondral lesions and osteoarthritis of the ankle, with no severe complications. The current scarcity of studies and their low-quality level preclude definitive conclusions presently.

**Level of evidence:**

III.

## Introduction

Mesenchymal stem cells (MSCs) have been isolated from bone marrow, periosteum, umbilical cord blood, dermis, infrapatellar fat pad, adipose tissue, synovium, skeletal muscles, and deciduous teeth [1]. These cells are multi-potent stem cells capable of differentiating into cells of connective tissue lineages. It is now commonly accepted that their action mechanism is mainly due to MSCs paracrine expression of a variety of bioactive factors acting with immunomodulatory and trophic fashion. Indeed, the patient's own resident stem cells construct the new tissue, stimulated by the bioactive factors secreted by the exogenously supplied MSCs [2]. The MSCs therefore may provide chondrogenic and chondroprotective capacity to arthritic joints [1, 3, 4]. For these reasons, MSCs have attracted attention as an ortho-biologic cellular therapy in regenerative medicine [5–8].

Although several sources from adult progenitor cells have been reported, in the last decade, adipose-derived mesenchymal stem cells (ADMSCs) have been recognized as an alternative source of stromal cells [9, 10]. Some studies [11–13] showed that ADMSCs have a chondrogenic potential similar to bone marrow derived MSCs, and moreover are easier to be obtained. As a matter of fact, subcutaneous stores in the infrapatellar fat pad and buttocks/flank allow for a less invasive harvesting process with lower donor site morbidity and lesser complications than the other stromal cells harvesting. Finally, lipoaspirate has been demonstrated to result in higher progenitor cell yields than bone marrow aspirates [11–15].

According to the isolation methods, three different categories of adipose-derived therapies can be identified: Adipose-derived stem cells (ADSCs), stromal vascular fraction (SVF), and micronized adipose tissue (MAT). The term ADSCs should be used when referring to MSCs isolated from adipose tissue and expanded in culture [16]. SVF typically requires centrifugation and collagenase enzymatic digestion procedures, where the cells are re-leased from their collagen matrix [17]. Mechanical separation of adipose tissue without using collagenase releases the cells from lipoaspirate, producing “micronized fat” (MAT) through minimal manipulation [18].

Despite the growing research on the role of ADMSCs therapy in osteoarthritis and cartilage repair, the scientific production has been less focused on the ankle.

The purpose of this systematic review was to analyze the current use in literature of ADMSCs in humans and presents the available evidence on their therapeutic potential in the treatment of ankle orthopedic issues, evaluating their applications and results.

## Materials and methods

### Search strategy

A review of the literature concerning the clinical applications of ADMSCs in ankle orthopedic pathologies was conducted independently by 2 of the authors (AA and EA) using PubMed, Google Scholar, EMBASE and Cochrane Library database on March 1, 2023. The search terms used were: “adipose derived stem cells”, “ankle”, “talus”. Field codes were used for database searches and each database was searched using the specific retrieve terms, and Medical Subject Headings (MeSH). The complete retrieve strategies were the following: ("ankle"[MeSH Terms] OR "ankle"[All Fields] OR "ankle joint"[MeSH Terms] OR ("ankle"[All Fields] AND "joint"[All Fields]) OR "ankle joint"[All Fields] OR "ankles"[All Fields] OR "ankle s"[All Fields] OR ("talus"[MeSH Terms] OR "talus"[All Fields])) AND (("adipose tissue"[MeSH Terms] OR ("adipose"[All Fields] AND "tissue"[All Fields]) OR "adipose tissue"[All Fields] OR "adipose"[All Fields] OR "adiposities"[All Fields] OR "adiposity"[MeSH Terms] OR "adiposity"[All Fields]) AND ("analogs and derivatives"[MeSH Subheading] OR ("analogs"[All Fields] AND "derivatives"[All Fields]) OR "analogs and derivatives"[All Fields] OR "derivatives"[All Fields] OR "de-rivable"[All Fields] OR "derivant"[All Fields] OR "derivants"[All Fields] OR "deri-vate"[All Fields] OR "derivated"[All Fields] OR "derivates"[All Fields] OR "deriva-tion"[All Fields] OR "derivations"[All Fields] OR "derivative"[All Fields] OR "derive"[All Fields] OR "derived"[All Fields] OR "derives"[All Fields] OR "deriving"[All Fields]) AND ("stem cells"[MeSH Terms] OR ("stem"[All Fields] AND "cells"[All Fields]) OR "stem cells"[All Fields])).

Reference lists of all included publications were checked for potential studies.

### Selection criteria

The PRISMA (Preferred Reporting Item for Systematic Reviews and Meta-Analyses) guidelines were followed, and a flowchart was used to summarize the selection procedure of the reviewed studies [19].

Inclusion criteria were determined and agreed upon between the reviewers. The inclusion criterium was the use of ADSCs in humans applied to bony orthopedic diseases of the ankle such as osteoarthritis and osteochondral lesions.

Exclusion criteria were non-English publications, review and meta-analyses articles, expert opinions and letter to Editor, animal studies and in vitro studies, participants under 18 years old, rheumatic diseases and septic ankle, the absence of clinical evaluation outcomes scores.

After duplicates removal, title and abstracts of all articles were screened for eligibility independently by 2 reviewers (AA and EA) and the papers of interest were selected for the full text. At full-text review, agreement of 2 reviewers was needed for study inclusion or exclusion. Disputes regarding inclusion of an article were resolved from the senior author (CF).

### Data abstraction and quality assessment

The included studies were analyzed by two reviewers to collect the following data according to PICO question (participants, intervention, comparisons, and outcomes):

- Authors, year of publication, study type and level of evidence (LOE).
- Participants: number of ankles, patients demographic characteristics (age, gender) and mean of follow up.
- Intervention: pathology, isolation methods, clinical applications, concomitant procedures.
- Comparisons: differences of clinical outcomes before and after the use of ADSCs.
- Outcomes: clinical outcomes through the PROMs, such as American Orthopaedic Foot and Ankle Society's (AOFAS), Foot and Ankle Outcome Score (FAOS), Foot and Ankle Disability Index (FADI), Tegner score and Visual analogue scale (VAS), and complications.

Data collection was performed using Microsoft Excel (Microsoft Corporation, Redmond, Washington, USA) for Windows 11.

Quality assessment of included studies was performed by two reviewers (A.A. and E.A.) independently using the Methodological index for non-randomized studies (MINORS) score [20].

### Data analysis

Information retrieved from the studies was reported with the use of descriptive statistics. Continuous variables were reported as mean value and standard deviation or range.

## Results

### Study selection

**Fig. 1:**
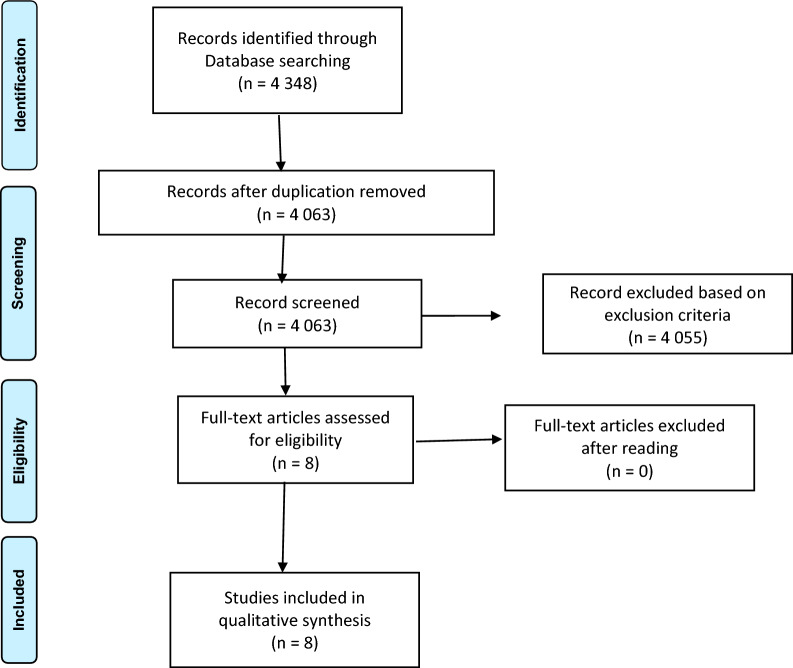
Flowchart of review process by PRISMA

The literature search yielded 4348 articles from Database search engine. After removing duplicates and reviewing all studies according to excluding criteria, 8 articles were identified for full-text review. After this evaluation, all 8 studies met the inclusion criteria and were included in the qualitative synthesis. The selection review process is summarized in Fig. 1.

All articles included were published between 2013 and 2021.

### Quality of evidence

The methodological quality assessment, as measured by the MINORS score, is summarized in Table 1. We considered the 8 items of MINORS score for non-comparative study of the eligible papers and the 12 items for comparative study design. The mean MINORS score was 7.3 for non-comparative study and 16.8 for comparative study.

**Table 1:** Quality assessment for included studies

References	Clearly stated aim	Inclusion of consecutive patients	Prospective collection of data	Endpoint appropriate to the study aim	Unbiased evaluation of endpoints	Follow-up appropriate to the study aim	Loss to follow-up less than 5%	Prospective calculation of the study size	Adeguate control group	Contemporary groups	Equivalence of groups	Adeguate statistical analyses	Total
Shimozono et al. [21]	2	2	0	2	1	1	2	0					9
Natali et al. [22]	2	2	2	2	1	2	1	0					11
Freitag et al. [23]	2	0	2	2	0	–	–	0					6
D'ambrosi et al. [24]	1	1	0	1	0	–	–	0					3
Kim et al. [25]	2	2	1	2	1	2	2	0	2	2	2	2	18
Kim et al. [26]	2	2	1	2	1	2	2	0	2	2	2	2	18
Kim et al. [27]	2	2	1	2	1	2	1	0	2	2	2	2	17
Kim et al. [28]	2	2	1	2	1	1	0	0	2	1	2	2	14

The items are scored 0 (not reported), 1 (reported but inadequate) or 2 (reported and adequate), thus the ideal global score for non-comparative studies is 16 and for comparative 24

### Population data

Patients’ characteristics are reported in Table 2. The total number of included ankles was 167. Six papers reported the gender distribution: in total 64 females (38%) and 79 males (47%) were included. When reported, the patient’s mean age was 49.2 ± 15.1 years (range 42–56.8). Mean follow-up (FU) was 21.4 months.

**Table 2:** Studies and patients’ characteristics

References	Study design	LOE	Number of ankles	Age	Gender	Mean FU (Months)
Shimozono et al. [21]	Retrospective cohort study	IV	19	49.2 ± 15.0	–	14.3
Natali et al. [22]	Prospective not randomized cohort study	II	31	51.0 ± 15.5	12F/19 M	24
Freitag et al. [23]	Case report	IV	1	42	M	24
D'Ambrosi et al. [24]	Video article-Case series	IV	4	–	–	6
Kim et al. [25]	Retrospective cohort study	III	31	52.2 ± 5.9	16F/15 M	27.6 ± 5.0
Kim et al. [26]	Retrospective cohort study	III	26	53.6 ± 5.6	15F/11 M	27.7 ± 2.4
Kim et al. [27]	Retrospective cohort study	III	24	48.6 ± 10.9	8F/16 M	27.8 ± 5.6
Kim et al. [28]	Retrospective cohort study	III	31	56.8	13F/17 M	20.1 ± 4.7

*LOE* Level of evidence; *FU* Follow-up; *M* Male; *F* Female

### Isolation method and clinical applications

The ADSCs isolated from adipose tissue and expanded in culture was utilized by only one study [21], moreover they were the lonely that injected ADSCs into the ankle joint through ultrasound guidance 3 times 6 months apart. The remaining studies performed injections on the same day of the arthroscopy after stem cell preparation. Four studies performed intra-articular ankle arthroscopic injection of autologous SVF [22–25], while the autologous MAT was prepared by 3 studies, 2 of them injected it into the ankle joint through arthroscopic fashion [26, 27], and one performed a closed intra-articular injection [28] (Table 3).

**Table 3:** Studies ‘intervention, outcomes, and complications

References	Pathology	Clinical application	Concomitant procedure	Outcomes pre-op	Outcomes post-op	Statistical significance	Complications
Shimozono et al. [21]	PTA with KL grade 3 (8 cases), 4 (11 cases)	Intra-articular ankle arthroscopic injection of autologous MAT	2 concomitant flowable calcium phosphate injection into subchondral cysts of the tibial plafond, 1 ankle ligament stabilization	FAOS: 43.6 ± 9.8 VAS: 6.6 ± 1.5	FAOS: 53.8 ± 13.5 VAS (6 months): 3.9 ± 1.9 31.5% unsatisfied	Pain & QoL subscales, and overall FAOS (*p* < 0.05) VAS at 6 months (*p* < .001)	None
Natali et al. [22]	PTA KL grade 1 (3 cases), 2 (15 cases), 3 (13 cases)	Closed intra-articular injection of autologous MAT	–	AOFAS: 56.4 ± 17.5 FADI: 59.4 ± 16.9 VAS:7.0 ± 0.9	AOFAS: 84.2 ± 12.2 (12 m) 66.5 ± 15.7(24 m) FADI: 82.0 ± 11.4 (12 m) 71.8 ± 10.8(24 m) VAS: 3.3 ± 0.6 (12 m) 4.3 ± 1.2 (24 m)	AOFAS, FADI, VAS (*p* < 0.05)	5 Patients (16%) transitory intra-articular burning sensation after the injection or mild articular pain for a few days- No severe side effects
Freitag et al. [23]	OCLT	Intra-articular ultrasound guidance injection of autologous ADMSCs	Prior arthroscopic excision and curettage of a focal OCLT	FADI: 61%	FADI: 91%	FADI (*p* < 0.05)	Transitory intra-articular burning sensation after the injection
D'Ambrosi et al. [24]	OCLT	Intra-articular ankle arthroscopic injection of autologous MAT	Concomitant arthroscopic microfracture	AOFAS: 46.7 VAS: 8	AOFAS: 83.75 VAS: 2.2	AOFAS, VAS (*p* < 0.05)	None
Kim et al. [25]	Medial ankle osteoarthritis and varus deformity	Intra-articular ankle arthroscopic injection of autologous SVF	Concomitant arthroscopic microfracture, Supramalleolar osteotomy	VAS: 7.2 ± 0.8 AOFAS: 61.0 ± 5.8	VAS: 3.7 ± 1.5 AOFAS: 85.2 ± 5.2	AOFAS and VAS (*p* < 0.001)	–
Kim et al. [26]	Medial ankle osteoarthritis and varus deformity	Intra-articular ankle arthroscopic injection of autologous SVF	Concomitant arthroscopic microfracture, lateral sliding calcaneal osteotomy	VAS: 7.4 ± 0.8 AOFAS: 63.5 ± 4.2	VAS: 3.1 ± 1.5 AOFAS: 84.2 ± 7.9	AOFAS and VAS (*p* < 0.05)	–
Kim et al. [27]	OCLT	Intra-articular ankle arthroscopic injection of autologous SVF	Concomitant arthroscopic microfracture, (7) lateral ligament reconstruction	AOFAS: 67.7 ± 4.7 VAS: 7.1 ± 0.8 Tegner score: 3.4 ± 0.5	AOFAS: 83.3 ± 7.0 VAS: 3.2 ± 0.8 Tegner score: 3.9 ± 0.7	AOFAS, VAS, Tegner score (*p* < 0.05)	–
Kim et al. [28]	OCLT	Intra-articular ankle arthroscopic injection of autologous SVF	Concomitant arthroscopic microfracture	AOFAS: 68.1 ± 5.6 VAS: 7.1 ± 1.0 Tegner score: 3.5 ± 0.7	AOFAS: 82.6 ± 6.4 VAS: 3.2 ± 0.9 Tegner score: 3.8 ± 0.7	AOFAS, VAS, Tegner score (*p* < 0.05)	–

*PTA* Posttraumatic osteoarthritis; *KL* Kellgren-Lawrence; *OCLT* Osteochondral lesions of the talus; *FAOS* Foot and ankle outcome score; *AOFAS* American orthopaedic foot and ankle society; *FADI* Foot and ankle disability index; *VAS* Visual analogue scale; *QoL* Quality of life

### Osteochondral lesions

Four studies [21, 24, 25, 27] dealt with osteochondral lesion of the talus (OCLT), see Table 3. Freitag et al. [21] conducted a case report where the 42 years old patient underwent to prior arthroscopic excision and curettage of a focal OCLT and sequentially 3 times intraarticular ultrasound-guided injection of autologous ADMSCs. FADI score showed significant improvement in pre- to postoperative time (*p* < 0.05). Moreover, MRI with additional T2 mapping techniques showed successful regeneration of hyaline-like cartilage.

In a case report by D'Ambrosi et al. [27] the AOFAS and VAS score recorded a significant improvement before and after intervention (*p* < 0.05).

Kim et al. [24] showed how clinical (AOFAS, VAS and Tegner score) and MRI outcomes after an SVF injection with marrow stimulation improved significantly from pre- to postoperative period (*p* < 0.05) and compared it with marrow stimulation alone.

Kim et al. [25] reported significant improvement in clinical outcomes, including AOFAS, VAS and Tegner score (*p* < 0.05), in patients over 50 years old with OCLT that had SVF injection with marrow stimulation. Moreover, the outcomes of this group were better compared to those of marrow stimulation alone, especially when the lesion size was larger than 109 mm^2^ or a subchondral cyst existed.

### Osteoarthritis

Post-traumatic osteoarthritis (PTA) was considered in four articles [22, 23, 26, 28] (Table 3). Shimozono et al. [26] divided PTA patients into 2 groups considering Kellgren–Lawrence (KL) classification, 8 patients were collected in grade 3 and 11 cases in grade 4. The outcomes, including FAOS and VAS, showed a significant improvement before and after intervention in all scores, but no significant change was noted for the FAOS subscales of daily activities and symptoms. The overall FAOS score demonstrated a more significant improvement in pre- to postoperative change for KL grade 3 group than KL grade 4 group (*p* = 0.048).

Natali et al. [28] included 3 patients in KL grade 1, 15 in grade 2 and 13 cases in grade 3. A statistically significant improvement from basal evaluation to the 6, 12-, and 24-month FU was observed for AOFAS, FADI and VAS, whereas a statistically significant worsening from the 12-month to the 24-month FU was recorded.

In 2016, Kim et al. [22, 23] conducted 2 comparative studies. In one paper ADMSC injection with marrow stimulation was compared to marrow stimulation alone in patients with varus ankle osteoarthritis who have undergone lateral sliding calcaneal osteotomy [23]. The other one compared ADMSC injection with marrow stimulation to marrow stimulation alone in patients with varus ankle osteoarthritis treated with supramalleolar osteotomy [22]. The clinical and second-look arthroscopic outcomes of ADMSC injection with marrow stimulation were better related to those of marrow stimulation alone in patients with varus ankle osteoarthritis treated with bony associated procedures.

### Concomitant procedure

In 5 papers the authors performed concomitant bone marrow stimulation through arthroscopic microfractures [22–25, 27], in one article a prior arthroscopic excision and curettage of a focal OCLT was made [21]. Other concomitant procedures were summarized in Table 3. Moreover, 2 studies performed bony procedures: lateral sliding calcaneal osteotomy [23] and supramalleolar osteotomy [22] to treat medial ankle osteoarthritis and varus deformity, associated to bone marrow stimulation and intra-articular ankle arthroscopic injection of autologous SVF.

### Complications

No severe side effects were recorded from all studies considering the injection site or the donor site. Natali et al. [28] reported in 5 patients (16%) transitory intra-articular burning sensation after the injection or mild articular pain for a few days. Similar symptoms were recorded by Freitag et al. [21].

## Discussion

This systematic review assessed the current literature on the clinical applications and results of ADMSCs in bony orthopaedic diseases of the ankle. Although the literature concerning the knee application of ADMSCs is wide, this paper represents the first systematic review concerning the application of ADMSCs on ankle joint.

### Isolation methods and clinical applications

ADSCs expansion step is essential to generate sufficient cell numbers and requires among 24–48 h of incubation [16]. Thus, ADSCs culture present some drawbacks: require a two-stage procedure before administration, are expensive to produce because requiring competent staff and specific laboratory equipment and require a regulatory approval. Moreover, the delivery of ADSCs alone is not sufficient to regenerate damaged cartilage, but if they are incorporated in biomaterial scaffolds with cytokine growth factors, led to a significant increase of proliferation cells and chondrogenic marker expression [29, 30].

Differently, SVF or MAT isolation require a one-step procedure, and are relatively cost saving, but SVF isolation at the point of care for immediate clinical administration has to comply with strict regulatory requirements [17]. On the other hand, MAT method generally is not associated with expensive equipment and can be readily used without regulatory issues of enzymatic manipulation and cell expansion [9, 10, 31, 32]. Additionally, MAT preserves the cell and tissue microarchitecture of adipose tissue and includes high numbers of pericytes cells with an intact functional extracellular matrix [18].

At once, however, no clinical evidence has demonstrated the effectiveness of one system over the others [33, 34] and the current literature is poor about comparison of the various available formulations.

As regards the administration methods and considering the same outcomes, the studies that carry out ultrasound-guided or closed administration reported some complications; however, this finding may be biased by the fact that other studies did not pay attention to or record minor complications.

### Osteochondral lesions

Osteochondral lesions seem to better respond to MAT injection than marrow stimulation alone, even in patients over 50 and in large lesions [24, 25].

Generally, significant improvement was recorded to clinical outcomes following cell injection. In the case report by Freitag [21] the patient reported persistent limitation in sporting pursuits and recreational activity, although the T2 mapping MRI showed successful regeneration of hyaline-like cartilage.

### Osteoarthritis

It is interesting to note that in one study [26] although the improvement of all outcomes, the AOFAS subscales of daily activities and symptoms did not record significant change. A possible explanation could be that, although MAT can improve patients’ pain, the improvement is not enough to allow them to return to their daily activities. Indeed, MAT injection improved the VAS at 6 months but an increasing in VAS was observed at final FU. Initial symptoms improvement followed by long-term gradual worsening may suggest that MAT therapy provides significant, but gradually decreasing, pain relief in ankle osteoarthritis.

A transient improvement was also observed by Natali et al. [28], who showed a significant worsening from the 12-month to the 24-month FU visit.

Hence, ADMCS therapy may represent a non-surgical option to treat degenerative joint ankle disease in order to postpone invasive procedures especially in younger patients.

### Concomitant procedure

Few prospective studies evaluated the benefit of ADMSC in isolated injections. Many studies observed that good outcomes were recorded when axis realignment of a varus deformity was performed [35, 36]. Therefore, it is difficult to determine the therapeutic effect of regenerative medicine when it is associated with bone procedures. Future studies could compare ADMSC injection alone to cell injection associated with bone procedures, in order to verify whether the effect is synergistic or indifferent.

### Complications

The studies included in this review reported no serious complications; however, in the literature the most common complications concern the donor site, such as infection and pain. But these were lower than traditional Bone Marrow-MSC harvesting [8].

### Limitation

A great limitation can be addressed to the type of studies included, with no randomized double-blinded trials or comparative studies, leading to a lack of a control to confirm the efficacy of ADMSCs. The quality of these studies was extremely poor: notably, four out of eight studies [22–25] were conducted by the same research team, and two studies [21, 27] are a case report.

Furthermore, in many studies ADMSCs injection was performed in association with other intraarticular injections or surgical procedures, such as debridement, marrow stimulations, bony procedures. Therefore, any clinical results are unable to be attributable solely to the ADSCs injection.

## Conclusion

Based on the current literature ADSC injection, especially through the nonenzymatic mechanical methods, looks to be simple and promising treatment, without severe complications, for osteochondral lesions and osteoarthritis of the ankle. The current scarcity of studies and their limited level of evidence preclude definitive conclusions presently. Nonetheless, the encouraging outcomes should stimulate further high-level trial studies in this field.
