# Correction to: Adipose‑derived stem cells applied to ankle pathologies: a systematic review

**DOI:** 10.1007/s12306-023-00809-7

**Published:** 2024-04-06

**Authors:** A. Arceri, A. Mazzotti, E. Artioli, S. O. Zielli, F. Barile, M. Manzetti, G. Viroli, A. Ruffilli, C. Faldini

**Affiliations:** 1 1st Orthopaedics and Traumatologic Clinic, IRCCS Istituto Ortopedico Rizzoli, Via Giulio Cesare Pupilli 1, 40136 Bologna, Italy; 2 Department of Biomedical and Neuromotor Sciences (DIBINEM), Alma Mater Studiorum University of Bologna, 40123 Bologna, Italy

**Correction to: MUSCULOSKELETAL SURGERY** 10.1007/s12306-023-00798-7

In the original version of this article, the affiliation details for Author S. O. Zielli were incorrectly given as

^1^1st Orthopaedics and Traumatologic Clinic, IRCCS Istituto Ortopedico Rizzoli, Via Giulio Cesare Pupilli 1, 40136 Bologna, Italy

but should have been

^1^1st Orthopaedics and Traumatologic Clinic, IRCCS Istituto Ortopedico Rizzoli, Via Giulio Cesare Pupilli 1, 40136 Bologna, Italy

^2^Department of Biomedical and Neuromotor Sciences (DIBINEM), Alma Mater Studiorum University of Bologna, 40123 Bologna, Italy

